# Effect of Vitamin D3 in combination with Omega-3 Polyunsaturated Fatty Acids on NETosis in Type 2 Diabetes Mellitus Patients

**DOI:** 10.1155/2021/8089696

**Published:** 2021-10-22

**Authors:** Liliya Yu. Basyreva, Tatyana V. Vakhrusheva, Zoya V. Letkeman, Dmitry I. Maximov, Evgeniya A. Fedorova, Оleg M. Panasenko, Evgeny M. Ostrovsky, Sergey A. Gusev

**Affiliations:** Federal Research and Clinical Center of Physical-Chemical Medicine of Federal Medical Biological Agency, Moscow 119435, Russia

## Abstract

An understanding of the consequences of oxidative/halogenative stress triggered by neutrophil activation is impossible without considering NETosis. NETosis, formation of neutrophil extracellular traps (NETs), is known to promote microthrombus formation and impair wound healing in type 2 diabetes mellitus (T2DM) patients. Therefore, there is a need to search for drugs and treatment approaches that could prevent excessive NET formation. We aimed to evaluate the effect of vitamin D3 in combination with omega-3 polyunsaturated fatty acids (vitamin D3/omega-3 PUFAs) on NETosis in T2DM patients with purulent necrotizing lesions of the lower extremities. Patients and healthy subjects had vitamin D3 deficiency. Patients received, beyond standard treatment, 6000 IU of vitamin D3 and 480 mg of omega-3 PUFAs, and healthy subjects 1000 IU of vitamin D3 and 240 mg of omega-3 PUFAs daily for seven days. Neutrophil activation in ex vivo blood by phorbol-12-myristate-13-acetate (PMA) was used as a NETosis model. The percentage of blood NETs relative to leukocytes (NET_background_) before vitamin D3/omega-3 PUFA supplementation was 3.2%-4.9% in healthy subjects and 1.7%-10.8% in patients. These values rose, respectively, to 7.7%-9.1% and 4.0%-17.9% upon PMA-induced NETosis. In addition, the leukocyte count decreased by 700-1300 per 1 *μ*L in healthy subjects and 700-4000 per 1 *μ*L in patients. For both patients and healthy subjects, taking vitamin D3/omega-3 PUFAs had no effect on NET_background_ but completely inhibited PMA-induced NET formation, though neutrophils exhibited morphological features of activation. Also, leukocyte loss was reduced (to 500 per 1 *μ*L). For patients on standard treatment alone, changes occurred neither in background NETs and leukocytes nor in their amount after PMA stimulation. The decreased ability of neutrophils to generate NETs, which can be achieved by vitamin D3/omega-3 PUFA supplementation, could have a positive effect on wound healing in T2DM patients and reduce the incidence and severity of complications.

## 1. Introduction

Neutrophils, which are the largest population of circulating leukocytes (~60% in adults), play a key role in cellular innate immunity. Neutrophil granules contain a rich antimicrobial “arsenal,” which allows these cells to efficiently destroy phagocytosed pathogens [1]. During degranulation, antibacterial proteins and enzymes (myeloperoxidase (MPO), lactoferrin, lysozyme, neutrophil elastase (NE), etc.) can also be partially secreted from the cell [1]. The functioning mainly of NADPH oxidase and MPO leads to the formation of reactive oxygen- and halogen-containing compounds, the so-called reactive oxygen species (ROS) and reactive halogen species (RHS), which are directly involved in the destruction of pathogens both inside and outside the cell [2]. Another antimicrobial mechanism of neutrophils, which was discovered relatively recently, has been termed NETosis [3].

In NETosis, neutrophils release the so-called neutrophil extracellular traps (NETs), which are tangles of decondensed chromatin fibers and attached bactericidal agents including MPO, NE, histones, and defensins. To date, multiple inducers of NET formation have been described, among which are bacteria, fungi, viruses, immune complexes, lipopolysaccharides, phorbol-12-myristate-13-acetate (PMA), etc. NETs allow neutrophils to destroy extracellular pathogens while causing minimal damage to the host cells [4].

Although ROS and RHS are useful to kill microbes, their excessive production (as it can occur upon neutrophil activation at the inflamed site) is unfavorable, since it causes damage to biologically important molecules and cellular and tissue structures of the host body, leads to oxidative/halogenative stress, and provokes the occurrence of inflammation-associated diseases [5–8]. NETosis contributes to these harmful processes, since, firstly, it is activated by ROS produced with the participation of NADPH oxidase [4, 9], and secondly, MPO, which catalyzes the formation of RHS [2, 7], is among NET components [10, 11]. It has been indeed repeatedly observed that excessive NET formation is linked to the development of autoimmune, cardiovascular, and endocrine diseases, including diabetes mellitus. In particular, our data have shown that in type 2 diabetes mellitus (T2DM) patients with purulent necrotizing lesions of the lower extremities, a more severe clinical course is associated with a higher blood NET level [12, 13]. NETosis promotes microthrombosis as well as causes delayed wound healing [9, 14, 15], which could have a negative effect on the healing process in T2DM patients.

It has been found that some medications can induce NETosis [16, 17]. Therefore, it is worth checking the effect of drugs on NETosis as well as searching for new therapeutic options that prevent excessive NET formation [17–19]. This is currently especially important, since it has been demonstrated that when infected with COVID-19, people show an increase in NET blood concentration, and comorbid diabetes mellitus is associated with a severe course of pneumonia and higher mortality [20, 21].

Vitamin D3 or omega-3 polyunsaturated fatty acid (omega-3 PUFA) supplements can promote wound repair, as shown in T2DM patients with purulent necrotizing foot lesions [22, 23]. What is more, this effect was accompanied by a decrease in the blood plasma level of lipid peroxidation products, an increase in plasma reduced glutathione, an important antioxidant in the body [22], and an increase in the plasma total antioxidant activity [23], which indicates the involvement of oxidative stress in the development of purulent necrotizing wounds in T2DM as well as shows the antioxidant effect of vitamin D3 and omega-3 PUFAs.

A large number of data are available on the effects of vitamin D3 and omega-3 PUFAs on the functioning of various components of the immune system [24, 25], but only two studies investigated the effect of vitamin D3 on NETosis. In one of them, vitamin D3 inhibited [26], whereas, in the other, it activated NETosis [27]. No studies have examined how NET formation in blood is affected by vitamin D3 in combination with omega-3 PUFAs (vitamin D3/omega-3 PUFAs). Since the wound character state and healing process depend on the activation status of neutrophils, in particular, their predisposition to form NETs, the present work is aimed at assessing the effect of oral intake of vitamin D3/omega-3 PUFAs on the blood NET level as well as on PMA-stimulated NETosis in the ex vivo blood samples from T2DM patients with purulent necrotizing lesions of the lower extremities.

## 2. Materials and Methods

### 2.1. Subjects

The study included healthy subjects (*n* = 4) and T2DM patients with purulent necrotizing lesions of the lower extremities (*n* = 14) hospitalized at the Center for Purulent Surgery and Regenerative Technologies of the Federal Research and Clinical Center of Physical-Chemical Medicine of Federal Medical Biological Agency of Russia (FRCC PCM). Both patients and healthy subjects enrolled in the study had vitamin D3 deficiency.

### 2.2. Experimental Design

All patients received standard treatment, including glycemia correction, antibacterial therapy (based on bacteriologic microflora results, antibiotic susceptibility testing, and resistance PCR results), and anticoagulant, detoxification, rheological, anti-inflammatory, and immunomodulating therapy. Patients with comorbid disease were consulted by an endocrinologist, a therapist, and other specialists. All patients underwent treatment of the purulent site by hydrosurgery with the Versajet II hydrosurgery system (Smith & Nephew Inc., USA) and ultrasonic wound debridement with the Sonoca-190 ultrasonic generator (Söring, Germany). Slowly granulating wounds were subjected to negative pressure therapy with the VivanoTec system (Hartmann, Germany). In addition to standard treatment, 8 patients were given an oral water-soluble form of vitamin D3 (6000 IU) and omega-3 PUFAs (eicosapentaenoic acid 288 mg, docosahexaenoic acid 192 mg) daily during the entire hospital stay. Healthy subjects received an oral water-soluble form of vitamin D3 (1000 IU) and omega-3 PUFAs (eicosapentaenoic acid 144 mg, docosahexaenoic acid 96 mg) daily for 7 days. The supplement doses were determined based on the results of preliminary experiments (data not shown). For patients, higher doses were required to produce the effect on NET formation.

Blood from healthy subjects was taken before and 1, 4, and 7 days after the beginning of vitamin D3/omega-3 PUFA supplementation. Blood from patients was taken at admission to the hospital and after 7 days of either standard treatment or taking vitamin D3/omega-3 PUFAs in addition to it.

Peripheral venous blood was collected into sodium citrate vacutainers (MiniMed, Russia). Biochemical and hematological blood analyses included, among others, erythrocyte sedimentation rate (ESR), glycated hemoglobin (HbA1c) level, baseline blood glucose level, creatinine, urea, aspartate aminotransferase (AST) and alanine aminotransferase (ALT) activities, and 25-hydroxyvitamin D3 level (measured by HPLC).

### 2.3. Analysis of Blood Smears

Standardized blood smears were prepared immediately after drawing the blood samples and after 2 h of blood incubation at 37°C in the presence or absence of 100 nM PMA (Sigma-Aldrich, USA). Blood smears were examined using a Motic B3 microscope (Motic Asia, Hong Kong). A quantitative assessment of NETs was performed as described previously [16]. Briefly, blood smears were stained by the Romanowsky method. NETs among 300-500 leukocytes were counted in the middle third part of the smear, and the NETs-to-leukocyte percentage ratio was calculated. The ratio in the collected blood is hereinafter designated as NET_background_, and it is designated as NET_PMA_ for blood incubated with PMA and NET_control_ for blood incubated without PMA. Blood leukocyte concentration was determined using a Mythic 18 flow hematology analyzer (Orphee, Switzerland) immediately after blood sampling (L_background_) and after 2 h of blood incubation at 37°C in the presence of 100 nM PMA (L_PMA_) or without PMA (L_control_).

### 2.4. Data Analysis

The statistical analysis of results was performed with STATISTICA 6.0 software (StatSoft Inc., USA). The statistical significance for differences between the groups was assessed using Student's *t*-test and Mann–Whitney *U*-test.

## 3. Results

All healthy subjects enrolled in the study were deficient in serum 25-hydroxyvitamin D3. Their hematological and biochemical parameters were inside the reference limits, except for this vitamin D3 metabolite. Blood parameters in patients are shown in Table 1. No significant differences were found in the measured parameters between patients who received or not vitamin D3/omega-3 PUFAs. Worthy of note is a marked deficiency of 25-hydroxyvitamin D3 in all patients (<17.1 ng/mL, with a normal range of 30-100 ng/mL).

### 3.1. Healthy Subjects

**Figure 1:**
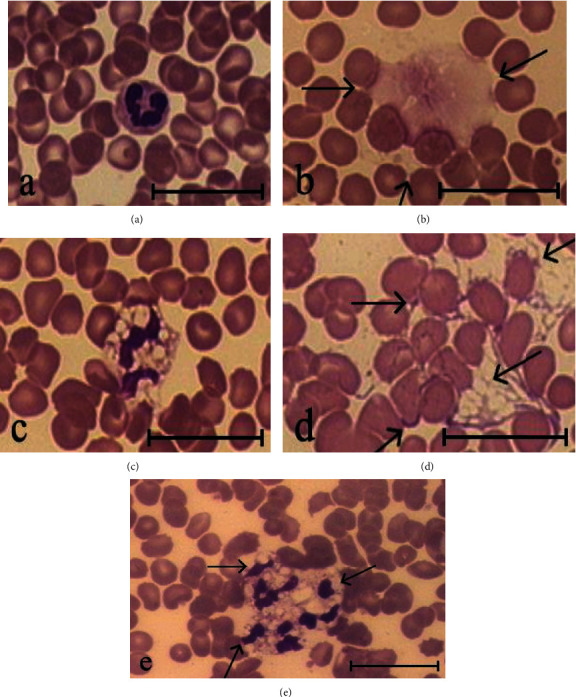
Neutrophils and NETs in blood smears from a healthy subject before vitamin D3/omega-3 PUFA supplementation. The blood was incubated (37°C, 2 h) with no added PMA (a, b) or with 100 nM PMA (c–e): (a) a resting neutrophil; (c) an activated neutrophil; (b, d) NETs (indicated by arrows); (e) a neutrophil aggregate (indicated by arrows). Scale bar: 20 *μ*m.

**Figure 2:**
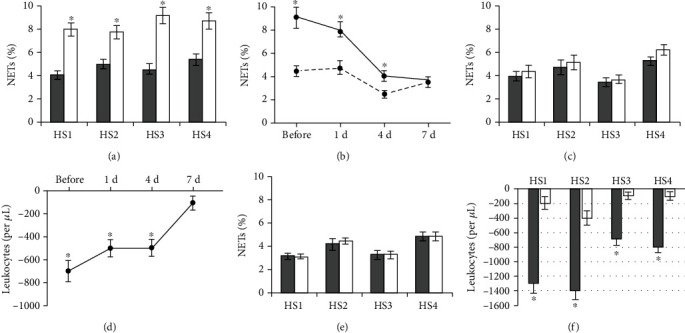
NETs and leukocytes in blood from healthy subjects (HS) before and after vitamin D3/omega-3 PUFA supplementation. Results of measurements done before (NET_background_, background NETs) or after blood incubation (37°C, 2 h) with no added PMA (NET_control_ and L_control_, control NETs, and control leukocytes, respectively) or with 100 nM PMA (NET_PMA_ and L_PMA_). ^∗^*P* < 0.001: (a) NET_control_ (■) and NET_PMA_ (□) before supplementation; (b) changes in NET_control_ (- - -) and NET_PMA_ (—) for 7 days of supplementation; (c) NET_control_ (■) and NET_PMA_ (□) after 7 days of supplementation; (d) changes in the difference between L_PMA_ and L_control_ for 7 days of supplementation; (e) NET_background_ before (■) and after (□) 7 days of supplementation; (f) the difference between L_PMA_ and L_control_ before (■) and after (□) 7 days of supplementation.

NETs in the blood of healthy subjects before the start of vitamin D3/omega-3 PUFA supplementation constituted 3.2% to 4.9% of the leukocyte population. We term NETs and leukocytes in the blood just after it collected the background NETs and leukocytes. Blood incubation for 2 h with 100 nM PMA resulted in neutrophil activation (Figures 1(a) and 1(c)) and NET formation, as evidenced by a significantly higher NET percentage (NET_PMA_) compared to control blood incubated with no added PMA (NET_control_) (Figure 2(a)). Among NETs formed during PMA-stimulated NETosis were those that differed from background NETs by the presence of fibrillar structures (Figures 1(b) and 1(d)), and also, there were aggregates consisting of 3–4 activated neutrophils (Figure 1(e)). The number of leukocytes in blood incubated with PMA (L_PMA_) was decreased by an average of 1050 (700-1400) per 1 *μ*L relative control blood samples (L_control_) (Figure 2(f)).

On the example of two healthy subjects, we found that with each day of vitamin D3/omega-3 PUFA intake, there was a gradual decrease in NET_PMA_ and the difference between L_PMA_ and L_control_ (Figures 2(b) and 2(d)). By day 7, NET_PMA_ and NET_control_ did not differ (Figure 2(b)), and the difference between L_PMA_ and L_control_ nearly disappeared (Figure 2(d)), and thus, we further used a 7-day time point to assess the effects of vitamin D3/omega-3 PUFA supplementation.

Healthy subjects' neutrophils exposed to PMA exhibited morphological features of activation; however, there were no aggregates of activated neutrophils. NET_PMA_ did not significantly differ from NET_control_ (Figure 2(c)), and the difference between L_PMA_ and L_control_ significantly decreased (Figure 2(f)). Vitamin D3/omega-3 PUFA supplementation had no effect on NET_background_ (Figure 2(e)).

### 3.2. Patients

**Figure 3:**
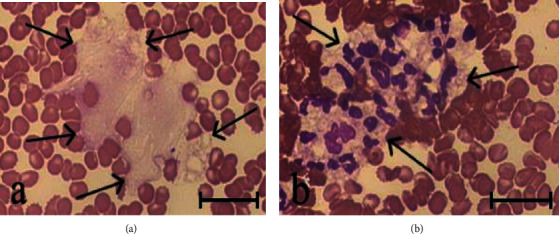
NET (a) and a neutrophil aggregate (b) in blood smears from a patient before vitamin D3/omega-3 PUFA supplementation. The blood was incubated (37°C, 2 h) with 100 nM PMA. Scale bar: 20 *μ*m.

**Figure 4:**
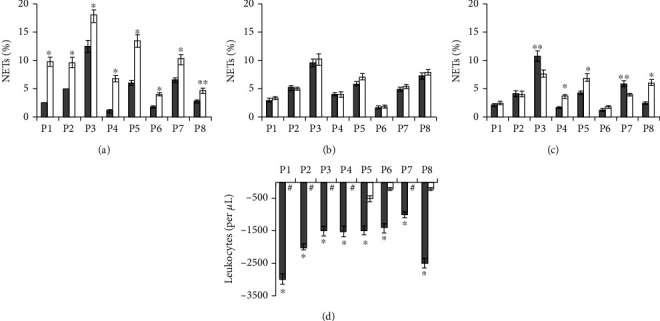
NETs and leukocytes in blood from patients (P) before and after vitamin D3/omega-3 PUFA supplementation in addition to standard treatment. Results of measurements done before (NET_background_, background NETs) or after blood incubation (37°C, 2 h) with no added PMA (NET_control_ and L_control_, control NETs, and control leukocytes, respectively) or with 100 nM PMA (NET_PMA_ and L_PMA_). ^∗^*P* < 0.001; ^∗∗^ − 0.01 < *P* < 0.05. (a) NET_control_ (■) and NET_PMA_ (□) before supplementation; (b) NET_control_ (■) and NET_PMA_ (□) after 7 days of supplementation; (c) NET_background_ before (■) and after (□) 7 days of supplementation; (d) the difference between L_PMA_ and L_control_ before (■) and after (□) 7 days of supplementation.

**Figure 5:**
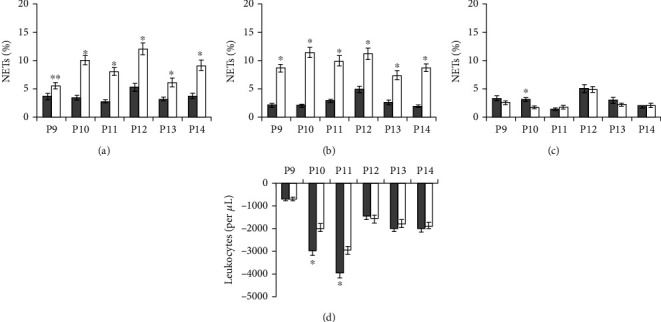
NETs and leukocytes in blood from patients (P) at admission to hospital and after 7 days of standard treatment alone. Results of measurements done before (NET_background_, background NETs) or after blood incubation (37°C, 2 h) with no added PMA (NET_control_ and L_control_, control NETs, and control leukocytes, respectively) or with 100 nM PMA (NET_PMA_ and L_PMA_). ^∗^*P* < 0.001; ^∗∗^ − 0.01 < *P* < 0.05. (a) NET_control_ (■) and NET_PMA_ (□) in patents before standard treatment alone; (b) NET_control_ (■) and NET_PMA_ (□) in patients after 7 days of standard treatment alone; (c) NET_background_ in patients before (■) and after (□) 7 days of standard treatment alone; (d) the difference between L_PMA_ and L_control_ in patients before (■) and after (□) 7 days of standard treatment alone.

No morphological signs of neutrophil activation were observed in blood smears taken from patients prior to treatment. The shape and size of background NETs and their affinity for dyes did not differ from those in healthy subjects. NET_background_ ranged from 1.7% to 10.8%. The blood incubation with PMA caused neutrophil activation and NET formation. Therewith, blood smears showed NETs whose size and structure differed not only from background NETs but also not rarely from NETs in PMA-treated blood from healthy subjects. These NETs were several times larger in area and were almost unstained (Figure 3(a)). The fibrillar filaments of great extension (into which the NETs in some cases disintegrated entirely) were clearly seen in them. There were also present large aggregates consisting of more than 10 activated neutrophils (Figure 3(b)), which significantly exceeded the size of aggregates observed in the healthy subjects' blood after incubation with PMA. Quantitative analysis of blood smears from patients before treatment showed that NET_PMA_ was significantly higher than NET_control_ (Figures 4(a) and 5(a)), and the difference between L_PMA_ and L_control_ was on average of 1750 (400-4000) per 1 *μ*L (Figures 4(d) and 5(d)).

#### 3.2.1. Patients on Standard Treatment plus Vitamin D3/Omega-3 PUFA Supplementation

For patients who were given vitamin D3/omega-3 PUFAs for 7 days along with the standard treatment, blood incubation with PMA led to neutrophil activation and the appearance of large aggregates of more than 10 activated neutrophils. Among NETs were the traps with an enlarged area and fibrillar structure, but NETs with a very large area and low affinity to the dye were rarely seen. NET_PMA_ was not significantly different from NET_control_ (Figure 4(b)). In five patients, there was no difference between L_PMA_ and L_control_, and the difference was minimal in three patients (Figure 4(d)). In addition, it should be noted an opposite direction in changes in NET_background_: three patients (P4, P5, and P8) after the course of vitamin D3/omega-3 PUFA use showed a significant increase in NET_background_, whereas two patients (P3 and P7) showed, on the contrary, a decrease (Figure 4(c)).

#### 3.2.2. Patients on Standard Treatment Alone

Analysis of blood smears of patients who received only the standard treatment showed that the pattern of PMA-induced neutrophil activation was similar to that observed prior to the start of treatment. Neutrophils had morphological features of activated cells, and large leukocyte aggregates were formed. NET_PMA_ remained significantly higher than NET_control_ (Figure 5(b)). The difference between L_control_ and L_PMA_ remained quite high, ranging from 500 to 3,000 per 1 *μ*L (Figure 5(d)). It should also be noted that NET_background_ significantly decreased in one patient (P10) and remained unchanged in the other five patients (Figure 5(c)).

## 4. Discussion

T2DM complicated by purulent necrotizing ulcers on the lower limbs is characterized by a long progressive course, which is associated with the development of vascular pathology and chronic nonhealing wounds with chronic inflammation. We have previously shown that the blood of mostly severe patients has high concentrations of NETs [12, 13].

Circulating NETs are known to contribute to clot formation and vascular endothelial cell damage [9, 14, 26]. Experimental data indicate that hyperglycemia promotes neutrophil priming/activation and NETosis [28, 29]. There are also published data showing that NETs in T2DM interfere with wound healing [15]. Thus, we can assume that NETs may play an important role in the development of T2DM complications.

Most studies aimed at investigating NETosis in T2DM were performed on experimental animal models or isolated neutrophils [15, 30, 31]. It is clear that the results of these studies were obtained under conditions significantly different from those for neutrophils in human blood.

Our approach to investigate NETosis is specific in that we use whole blood. This allows us to analyze the response of all neutrophils, while some fraction of neutrophils can be lost during isolation. Moreover, the cell loss during isolation can depend on whether the blood is from healthy individuals or patients with severe pathology. This makes it difficult to compare the data obtained for healthy subjects and patients. In addition, manipulations during the isolation procedure may affect neutrophils. Moreover, neutrophils being isolated found themselves out of their blood noncellular and cellular environment that significantly influences neutrophil behavior [32].

Quantification of both NETs and leukocytes in the whole blood after PMA-stimulated activation not only reveals the capacity of neutrophils to undergo NETosis but also controls neutrophil loss due to cell death, mainly by autophagy [33]. This allows a more adequate understanding of neutrophil's potential of activation in the blood vessel or at the tissue injury site.

The present study demonstrates that PMA-induced neutrophil activation in blood samples from T2DM patients with purulent necrotizing lesions of the lower extremities leads to NET formation and the appearance of neutrophil aggregates of much larger size than in healthy volunteer blood. This fact suggests that neutrophil activation in the blood circulation in T2DM patients can result, with high probability, in blood-flow blockage by large neutrophil aggregates, even in vessels of relatively large diameters, and, accordingly, in the formation of extensive areas deprived of normal blood supply. Taking into account also the assumption that NETs can accumulate in the branching capillary network [34], large-sized NETs should block blood flow in capillaries, additionally restricting blood supply to tissues.

In addition to having the potential to contribute to microcirculatory disorders, neutrophils play dual roles in the wound repair process. Rapid wound healing is associated with proper performance of the neutrophil program. The program goes through a phase of neutrophil accumulation to phagocytose and kills pathogens at the damage/infection site. However, excessive neutrophil retention in the wound as well as the presence there of toxic products released from necrotic neutrophils leads to delayed wound healing. Proper clearance of neutrophils is believed to be carried out either by macrophage engulfment of apoptotic neutrophils or due to reverse neutrophil migration back from sites of tissue injury into the vasculature [35]. Macrophage phagocytosis of apoptotic neutrophils is part of the normal resolution of inflammation.

Thus, the proper clearance of neutrophils implies that activated neutrophils after they have completed their functions at the inflamed site do not die by mechanisms other than apoptosis, e.g., NETosis, autophagy, or necrosis.

Our results show that taking vitamin D3/omega-3 PUFAs completely “switches off” NETosis and death of neutrophils upon their activation with PMA in whole blood ex vivo. This suggests that upon vitamin D3/omega-3 PUFA supplementation, neutrophil death in the wound site will not occur through NETosis, autophagy, or otherwise, which increases the possibility of neutrophil clearance by reverse migration into the bloodstream or macrophage phagocytosis. This should support faster wound repair.

The data reported in the literature suggest that vitamin D3/omega-3 PUFA supplement can significantly reduce ROS/RHS generation and increase the total antioxidant capacity of blood serum [22, 23, 36, 37] and that the prevention of NET formation enhances neutrophil phagocytic activity [38]. All these may potentially favor wound healing in T2DM patients with purulent necrotizing injuries on the lower limbs.

It should be noted that the ending background level of NETs in T2DM patients after 7 days of vitamin D3/omega-3 PUFA intake remained the same as the starting level or changed in opposite directions. Probably, the background NET formation was not blocked by a vitamin D3/omega-3 PUFA dosage used. It may be possible that background NETosis can be reduced by an increase in the dose and/or supplement-taking interval. However, it cannot be ruled out that there is a NETosis-inducing mechanism that remains active despite taking the supplement. Further studies should answer questions regarding supplement dose and course-length as well as the inducers and mechanisms of background NET formation.

It seems interesting to carry out analogous measurements also in T2DM patients who caught COVID-19, since T2DM in COVID-19 patients is associated with more severe pneumonia and higher mortality compared to nondiabetic subjects [21]. Sera from COVID-19 patients have been shown to have elevated levels of cell-free DNA, MPO/DNA complex, and citrullinated histone H3 (Cit-H3), which are NETosis specific markers. Importantly, both cell-free DNA and MPO/DNA complex were higher in hospitalized patients who were on a ventilator compared with patients with no need for lung ventilation. Finally, sera from people with COVID-19 showed an ability to induce NET formation in control neutrophils *in vitro* [39].

Some T2DM patients in our study displayed high background NET levels (P3, P7) (Figure 4(c)), while others with lower levels showed a higher level of neutrophil activation in response to PMA (P1, P4, P10, P11, and P14) (Figures 4(a) and 5(a)).

If we assume that NET formation provoked by COVID-19 infection is superimposed on the T2DM-mediated increase in NETs and neutrophil activation, it will lead to extremely high levels of circulating NETs and NET accumulation in the capillaries of lungs and other organs, causing reduced blood flow in the microcirculatory bed and thereby causing a more severe course of pneumonia and development of multiple organ failure.

Complete blocking of NET formation with vitamin D3/omega-3 PUFAs in T2DM patients, as we expect, should promote a more favorable course of pneumonia in the case of COVID-19 infection. The idea that blood NET levels and the degree of neutrophil activation can be considered predictors of pneumonia severity and risk of developing multiple organ failure in COVID-19 infection is also noteworthy. Taking into account the fact that vitamin D3/omega-3 PUFAs block NET formation not only in T2DM patients but also in healthy subjects, the use of this supplement should be considered a potential preventive measure and/or adjuvant therapy against COVID-19. This assumption is indirectly supported by the data that higher vitamin D3 levels in the population correspond to a significantly lower incidence rate of COVID-19 disease [40]. Clearly, these assumptions need to be tested in a double-blind, placebo-controlled study.

**Figure 6:**
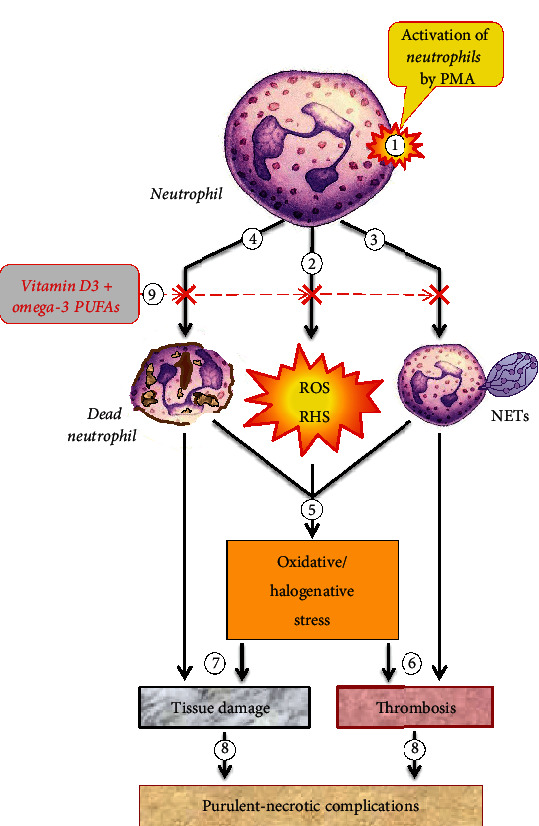
Summary scheme for the effect of vitamin D3/omega-3 PUFAs on T2DM complications such as purulent necrotizing ulcers, which may arise from neutrophil activation, NETosis, and oxidative/halogenative stress. Explanations are in the text.

Figure 6 gives a schematic summary of the data discussed above. Neutrophil activation (1 in Figure 6) leads to ROS/RHS production (2 in Figure 6), NET formation (3 in Figure 6), and cell death (4 in Figure 6). All these processes contribute to the development of oxidative/halogenative stress (5 in Figure 6) and can cause vascular thrombosis (6 in Figure 6), tissue damage (7 in Figure 6), and, as a consequence, delay of purulent necrotizing wound healing (8 in Figure 6), thereby considerably aggravating the disease course. Administration of an appropriate dosage of vitamin D3/omega-3 PUFAs reduces ROS/RHS generation, “switches off” the ability of neutrophils to form NETs, and prevents cell death (9 in Figure 6). This should reduce the likelihood and/or intensity of oxidative/halogenative stress, microcirculation alteration, and tissue damage, thereby reducing the severity or frequency of complications in diseases associated with impaired neutrophil function.

## 5. Conclusions

The intake of vitamin D3/omega-3 PUFAs in the dosage used in this study prevented PMA-stimulated NET formation and cell death in whole blood samples from both healthy subjects and T2DM patients. These changes in neutrophils should reduce the likelihood and/or intensity of oxidative/halogenative stress, prevent thrombosis in the microvasculature, and reduce the severity and frequency of complications in T2DM. In the future, it seems reasonable to assess the extent to which the possibility to regulate neutrophil function with vitamin D3/omega-3 PUFAs can be used to reduce the severity or frequency of complications in diseases associated with impaired neutrophil function. This pilot study, which included only a small number of patients and healthy subjects, should be followed by a double-blind placebo-controlled study of a sufficiently large number of participants.

## Figures and Tables

**Table 1:** Hematology and biochemistry parameters in patients who received standard treatment alone and patients who additionally received vitamin D3/omega-3 PUFAs. Displayed are median values with minimum and maximum values in parentheses. ND: no statistically significant difference between groups.

Parameters	Patients on standard treatment	Patients on standard treatment+vitamin D3/omega-3 PUFAs	Significant difference
Age (years)	69 (58, 80)	59 (50, 89)	ND
Gender (M/F)	3/5	3/3	ND
HbA1c (%)	8.8 (7.4, 14.8)	9.2 (8.4, 13.1)	ND
Baseline blood glucose (mmol/L)	9.6 (2.3, 17.5)	7.5 (5.0, 16.3)	ND
25-Hydroxyvitamin D3 (ng/mL)	7.3 (7.0, 17.1)	7.8 (5.1, 12.0)	ND
ESR (mm/h)	49 (8, 59)	42 (2, 60)	ND
ALT (U/L)	17.6 (6.2, 51.8)	11.3 (8.0, 27.3)	ND
AST (U/L)	20.1 (10.2, 26.9)	19.5 (10.2, 35.0)	ND
Creatinine (*μ*mol/L)	84.9 (78.4, 133.3)	107.1 (57.1, 179.6)	ND
Urea (mmol/L)	7.3 (3.7, 10.4)	7.3 (3.2, 10.7)	ND
Leukocytes (×10^9^/L)	9.8 (7.1, 31.4)	10.0 (5.6, 17.7)	ND

## Data Availability

The data used to support the findings of this study are included within the article. Additional information may be obtained from the corresponding author upon request.
